# Social comparison of ability and fear of missing out mediate the relationship between subjective well-being and social network site addiction

**DOI:** 10.3389/fpsyg.2023.1157489

**Published:** 2023-06-26

**Authors:** Philipp Steinberger, Hyunji Kim

**Affiliations:** ^1^Faculty of Psychology, University of Vienna, Vienna, Austria; ^2^Faculty of Psychology, Utrecht University, Utrecht, Netherlands

**Keywords:** subjective well-being, social comparison of ability and opinion, fear of missing out, SNS addiction, mediation

## Abstract

As social network sites (SNS) gain more users, the problem of unhealthy user behavior such as SNS addiction arises. We conducted a cross-sectional study (*n* = 296) on how subjective well-being (SWB) relates to SNS addiction by investigating two possible mediators: social comparison and the fear of missing out (FOMO). While doing so, we tested two distinct associations of social comparison: social comparison of ability (SCA) and social comparison of opinion (SCO). Splitting two components of social comparison is important because, while SCA involves social outcomes often depicted in SNS posts (e.g., performance, material wealth, health, and achievements) that might evoke negative emotions such as FOMO and jealousy, SCO involves presenting or sharing one’s beliefs and values in SNS posts (e.g., arguments, comments, and statements) that might evoke relatively little negative emotions. Our results showed that we replicated previous findings by demonstrating that social comparison and FOMO jointly mediated the relationship between SWB and SNS addiction. More importantly, SCA (together with FOMO), but not SCO, uniquely mediated the relationship between SWB and SNS addiction. Such distinct relations call for future research on identifying specific elements of social comparison contributing to the relation between FOMO and SNS addiction.

## 1. Introduction

Social network sites (SNS) provide a possibility to connect with people from all over the world, which is even more relevant in times of a worldwide pandemic where social contact is limited (Nabity-Grover et al., 2020; Tuck and Thompson, 2021). However, with the rise of SNS like Facebook, Instagram, Snapchat or TikTok, together with an ever-increasing number of SNS users, problematic behaviors emerge. In particular, excessive usage of SNS can develop into a form of addiction toward engaging in SNS activities also termed as SNS addiction (Andreassen and Pallesen, 2014). SNS addiction occurs when users are obsessively concerned about SNS, with a strong motivation to use SNS and invest excessive time and effort online, thereby negatively impacting important life domains such as social activities, work, and relationships, mental health and well-being (Andreassen and Pallesen, 2014; Donnelly and Kuss, 2016; Kuss and Griffiths, 2017; Moqbel and Kock, 2018; Reer et al., 2019). Kuss and Griffiths (2011) argued that SNS addiction shares six components identical to other behavioral addictions, namely mood modification (a change in emotional states through engagement in SNS), tolerance (time spent on SNS is increasing), salience (being preoccupied with SNS), conflict (interpersonal and intrapsychic problems emerge because of SNS usage), withdrawal symptoms (restricted SNS use leads to unpleasant physical and emotional symptoms), and relapse (reverting to excessive SNS usage after being on a period of abstinence). SNS addiction has been associated with numerous negative outcomes, such as lower subjective well-being (Satici, 2019), depression (Steers et al., 2014; Li, 2019), anxiety (Lee, 2014), low self-esteem (Midgley et al., 2021), and loneliness (Marino et al., 2018; Satici, 2019).

Of our interest, subjective well-being (SWB), defined as the level of well-being individuals experience in relation to their subjective evaluations of their lives (Diener and Ryan, 2009), can be highly affected by SNS activities because positive or negative life events involve fluctuating feelings and judgements about satisfaction with life, work, relationships, health, among other important domains that can be easily portrayed in SNS posts (Yang, 2020). On the one hand, SNS activities such as posting and looking through others’ life events modulate how the SNS users evaluate their own lives, on the other hand, dissatisfaction with life might trigger SNS users to engage in more SNS activities as an easy way to boost their own well-being (Satici and Uysal, 2015; Chen et al., 2016). This bidirectional relationship between SWB and SNS use might turn into a cyclic relationship, triggering constant social comparison processes which also severely affect users’ SWB (Chen et al., 2016) and self-esteem (Kim et al., 2021).

Frequently used SNS platforms such as Instagram, Facebook or TikTok allow users to present themselves positively and share rewarding experiences mainly *via* visual contents. The possibility to positively present oneself may evoke various social comparison processes in users to compare whether their lives are “better” than that of others (Lin and Utz, 2015). On the one hand, when viewing someone’s posts or profiles that are worse off, SNS users might feel more content about their own life which increases their own SWB and self-esteem (Kim et al., 2021). Such social comparison processes may lead to an increase in SNS use directly. But on the other hand, viewing others’ contents that showcase more rewarding experiences might induce feelings that one is missing out on something from which one feels excluded (Przybylski et al., 2013), namely fear of missing out (FOMO). These feelings may contribute to the desire to constantly stay in contact with others and keep informed about other people’s lives and activities (Przybylski et al., 2013). Due to excessive positive presentations prevalent in SNS platforms, FOMO can be triggered, and as a result, one’s satisfaction with life, general mood, and SWB might be worsened, while SNS engagement and SNS addiction are strengthened (Przybylski et al., 2013; Al-Menayes, 2016; Stead and Bibby, 2017; Yin et al., 2021). In the end, in order to boost one’s satisfaction with life, one may turn their eyes to SNS platforms again for an easy way out to escape the feelings of dissatisfaction, reinforcing a vicious circle of behavior, leaving open the possibility of developing SNS addiction (Burnell et al., 2019; Reer et al., 2019). In the present study, we argue that this vicious circle might be one explanation of the uncontrollable SNS use and the association might be stronger depending on “what” SNS users compare themselves on, when engaging in online social comparisons.

Social comparison generally serves the purpose of self-evaluation by comparing oneself with others’ abilities and opinions (Festinger, 1954). The existing literature has shown that engaging in social comparisons is positively associated with increased SNS usage (Ozimek and Bierhoff, 2016; Reer et al., 2019). Individuals who report low in SWB also engage more in social comparison processes to reduce self-uncertainty (Gibbons and Buunk, 1999; Butzer and Kuiper, 2006; Reer et al., 2019). Nevertheless, previous findings have so far largely focused on social comparison activities in general (Burnell et al., 2019; Reer et al., 2019), when social comparison activities are comprised of two distinct aspects depending on “what” is being compared: ability and opinion (Gibbons and Buunk, 1999). SNS posts provide ample materials for users to engage in social comparisons of abilities such as performance, physical appearance, material wealth, or achievements which are often disproportionately positively portrayed on SNS platforms (e.g., Saiphoo and Vahedi, 2019). Such posts might induce negative feelings in users because the comparison targets are frequently seen as performing better than themselves (Tandoc et al., 2015). In contrast, engaging in social comparisons of opinions might induce relatively little negative feelings because the comparison targets are not necessarily seen better or worse but seen as having the same or different opinions. Indeed, recent study has shown that SNS users with a higher ability comparison orientation experienced more emotions that led to lower life satisfaction like depression and envy, whereas SNS users with a higher opinion comparison orientation were less likely to experience these emotions and therefore reported higher life satisfaction (Park and Baek, 2018). Yet, this is the first evidence that needs further empirical validation.

**Figure 1:**
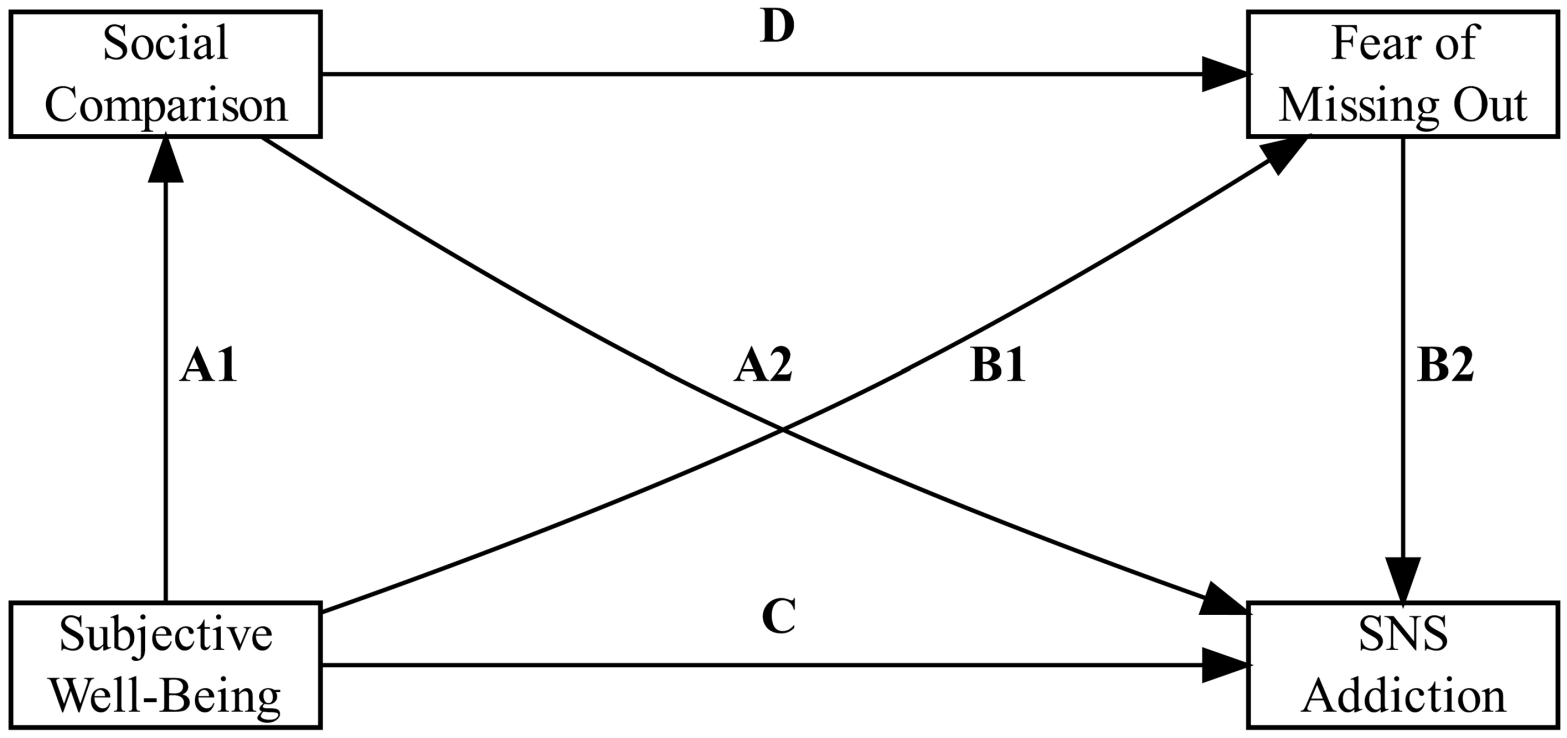
Proposed serial mediation model.

In the present study, we first examined whether the relation between SWB and SNS addiction can be explained by the tendency to engage in social comparisons and the fear of missing out (FOMO) in a serial mediation model (see Figure 1). Recently, Reer et al. (2019) demonstrated that the relationship between SWB and the tendency to engage in SNS activities was explained by the general tendency for engaging in social comparisons and FOMO. Based on this finding, we predicted that social comparison tendencies and FOMO will also jointly mediate the relation between SWB and SNS addiction as the bidirectional nature of SWB and SNS might propel the development of SNS addiction. After testing the first model, we separately tested social comparison of ability (SCA) and social comparison of opinion (SCO) measures consecutively as models 2 and 3.

Based on our rationale, our hypotheses are as follows.

*H*1: The negative relationship between SWB and SNS addiction is jointly mediated by engagement in social comparison and FOMO.

*H*2: SCA, but not SCO, jointly mediate the relation between SWB and SNS addiction with FOMO.

These main hypotheses are summarized in the model (see Figure 1). To test our hypotheses, we tested three models by integrating and separating SCA and SCO components of the social comparison measure (model 1: SCA and SCO, model 2: SCA, and model 3: SCO). The present study was conducted in accordance with the ethics guidelines of the Department Review Board of the University of Vienna.

## 2. Methods

### 2.1. Participants

Sample recruitment was conducted through the SNS platforms such as WhatsApp, Facebook, Instagram, and Reddit, as well as the survey platforms survey-circle.com and poll-pool.com. Participants reported that they were 18 years or older and completed the question “Do you use Social Media?” with “Yes.” To determine the required sample size, an *a-priori* Monte Carlo power analysis was conducted by using the mean standardized coefficients from Reer et al. (2019), since their combined model was similar to ours. Using a Monte Carlo simulation, we calculated the minimum sample size as *n* = 260 to achieve a power of .80 for the serial mediation (𝛼 = 0.05). The overall sample consisted of 378 participants. After excluding incompletely answered questionnaires (*n* = 60), incorrect attention check (Please select “strongly agree”) answers (*n* = 10) and outliers (*n* = 12) due to violations of mahalanobis distance, leverage and Cook’s distance, the final sample size consisted of 296 participants (M_age_ = 26.99 years, SD_age_ = 7.33) of which 72.97% identified as female (n = 216). Only four participants had compulsory education (Pflichtschulabschluss, i.e., middle school diploma), 15 had vocational education (Lehrabschluss, i.e., apprenticeship diploma), 107 had high school education (Matura, i.e., high school diploma) and 170 had a university degree.

### 2.2. Materials

#### 2.2.1. Subjective well-being

For measuring SWB, the German translated version of the 5-item Satisfaction with Life Scale (SWLS) was used (Diener et al., 1985; Janke and Glöckner-Rist, 2014). Participants indicated to what extent they agreed with each item raging from 1 (*strongly disagree*) to 7 (*strongly agree*).

#### 2.2.2. Social comparison

The Iowa-Netherlands Comparison Orientation Measure (INCOM) by Gibbons and Buunk (1999) is widely used for measuring both SCA and SCO. For this study, the shortened German version of 6-item INCOM by Schneider and Schupp (2011) was used. Participants indicated to what extent they agreed with each item given a scale ranging from 1 (strongly disagree) to 5 (strongly agree). Items 1, 2 and 3 served to measure SCA and items 4, 5 and 6 served to measure SCO.

#### 2.2.3. Fear of missing out

To measure FOMO, the German adaptation of the 10-item Fear of Missing Out Scale (FOMOS) was used (Przybylski et al., 2013; Spitzer, 2015). It consists of 10 items ranging from 1 (strongly disagree) to 5 (strongly agree).

#### 2.2.4. SNS addiction

The German adaption of the Bergen Social Networking Addiction Scale (BSNAS) was used to measure SNS addiction, which gauges the propensity of SNS addiction toward various SNS platforms in general (Andreassen et al., 2012; Kim et al., 2021). It consists of six items ranging from 1 (very rarely) to 5 (very often).

## 3. Results

### 3.1. Demographics and model fits

**Figure 2:**
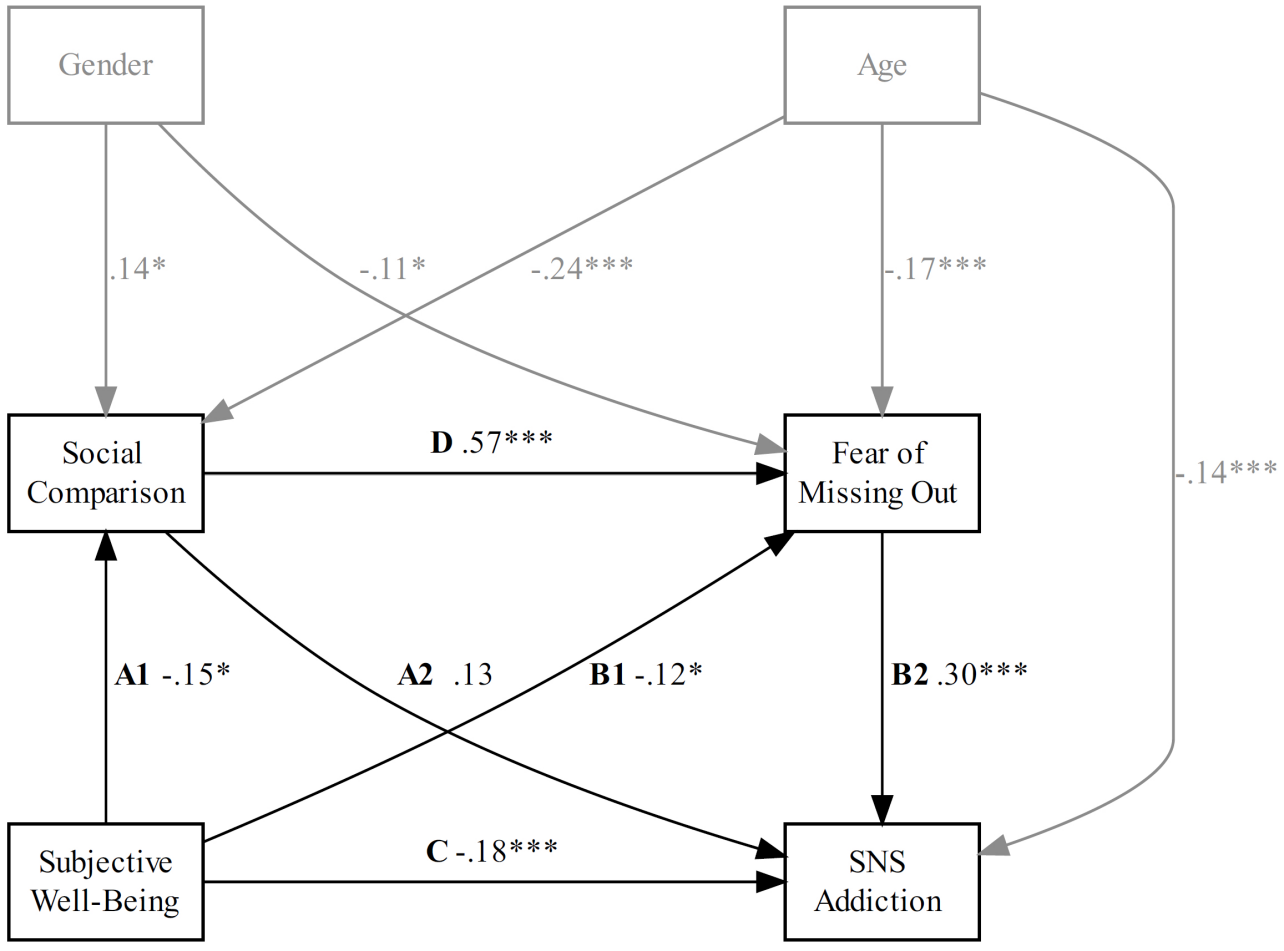
Model 1: estimated serial mediation. Path weights are standardized betas. *p < 0.05; **p < 0.01; ***p < 0.001. Model fit: CMIN/df = 1.124, TLI = 0.995, CFI = 1, RMSEA = 0.02, 90% CI = [0, 0.157].

**Figure 3:**
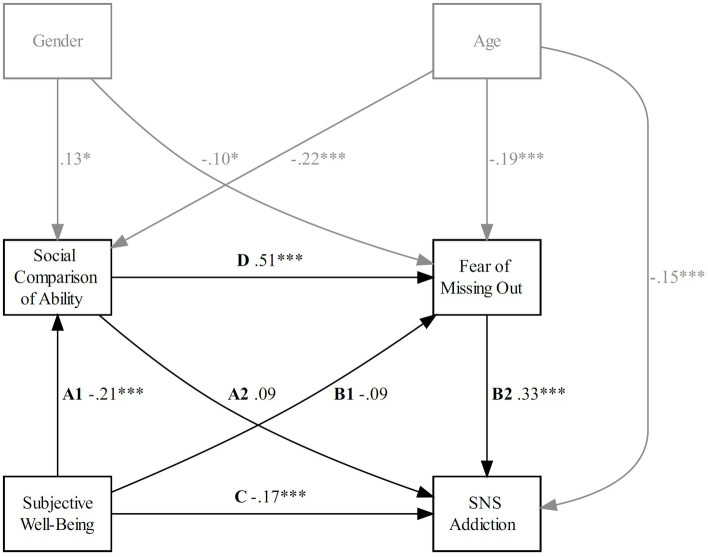
Model 2: estimated serial mediation. Path weights are standardized betas. *p < 0.05; **p < 0.01; ***p < 0.001. Model fit: CMIN/df = 1.474, TLI = 0.978, CFI = 0.998, RMSEA = 0.04, 90% CI = [0, 0.166].

**Figure 4:**
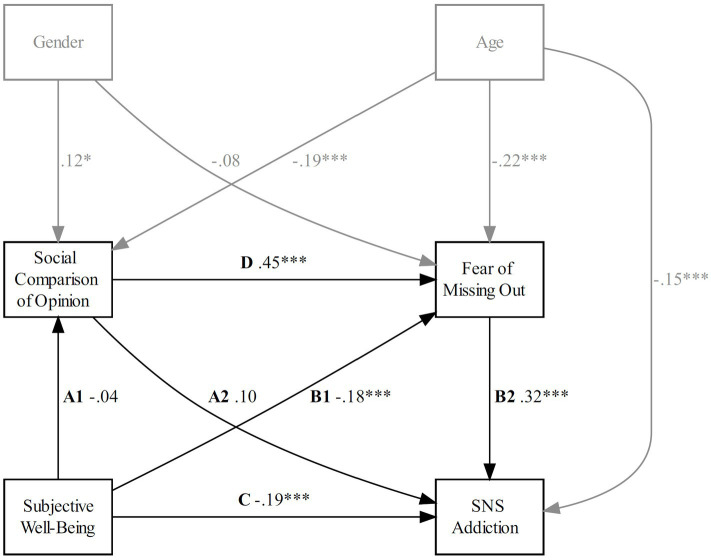
Model 3: estimated serial mediation. Path weights are standardized betas. *p < 0.05; **p < 0.01; ***p < 0.001. Model fit: CMIN/df = 1.421, TLI = 0.977, CFI = 0.998, RMSEA = 0.038, 90% CI = [0, 0.165].

Data was analyzed with R (Version 4.1.0) (R Core Team, 2021). Demographic information and intercorrelations are reported in Table 1. To test H1 and H2, three different mediation models were calculated. Paths were tested using bootstrapping (5,000 samples) with 90% bias-corrected confidence intervals (CI). Based on previous research, age served as a control variable for social comparison, SCA, SCO, FOMO, and SNS addiction, while gender served as a control variable for social comparison, SCA, SCO and FOMO (Przybylski et al., 2013; Ozimek and Bierhoff, 2016; Kuss and Griffiths, 2017; Reer et al., 2019). The fit of the three models was tested by calculating the following fit-indices: Tucker-Lewis index (TLI) >0.95, comparative fit index (CFI) >0.95, and root mean square error of approximation (RMSEA) <0.06 (Hu and Bentler, 1999). All three models showed a very good fit (see Figures 2–4).

**Table 1:** Demographic information and intercorrelations of measures.

Measures	*M*	SD	1	2	3	4	5	6	7
1. SWLS	25.03	6.02	(0.88)						
2. INCOM	17.83	5.05	−0.15*	(0.80)					
3. SCA	8.47	2.92	−0.22***	0.86***	(0.73)				
4. SCO	9.36	2.95	−0.04	0.86***	0.48***	(0.78)			
5. FOMOS	24.79	6.71	−0.21***	0.61***	0.56***	0.49***	(0.78)		
6. BSNAS	13.49	4.84	−0.27***	0.38***	0.35***	0.30***	0.46***	(0.76)	
7. Gender	0.73	0.44	0.04	0.15*	0.13*	0.12*	−0.03	0.06	
8. Age	26.99	7.33	0.03	−0.25***	−0.23***	−0.20***	−0.31***	−0.28***	−0.03

*p < 0.05; **p < 0.01; ***p < 0.001. Internal validities (Cronbach’s alphas) are reported in the diagonal.

### 3.2. Mediation analyses

H1 predicted that the negative relationship between SWB and SNS addiction is jointly mediated by engagement in social comparison and FOMO. As Figure 2 shows, lower SWB was significantly related to higher engagement in social comparison (Path A1) for Model 1 (𝛽 = −0.15, *p* = 0.011). The relationship between social comparison and SNS addiction (Path A2) was not significant for Model 1 (𝛽 = 0.13, *p* = 0.054). Higher engagement in social comparison was positively related to higher FOMO (Path D) for Model 1 (𝛽 = 0.57, *p* < 0.001). Lower SWB was associated with higher FOMO (Path B1) for Model 1 (𝛽 = −0.12, *p* = 0.012). Higher FOMO was significantly related to higher SNS addiction (Path B2) for Model 1 (𝛽 = 0.30, *p* < 0.001). The relationship between SWB and SNS addiction (Path C) was negative for Model 1 (𝛽 = −0.18, *p* < 0.001).

As Table 2 shows, the indirect effect 1 (Path A1 × Path A2) was not significant for Model 1 (𝛽 = −0.02, CI = [−0.04, 0.00], *p* = 0.129). The indirect effect 2 (Path B1 × Path B2) was significant for Model 1 (𝛽 = −0.03, CI = [−0.06, −0.01], *p* = 0.032), indicating that lower SWB was associated with higher SNS addiction. In support of H1, the indirect effect 3 (Path A1 × Path D × Path B2) was significant for Model 1 (𝛽 = −0.02, CI = [−0.04, −0.01], *p* = 0.033), indicating that lower SWB was associated with higher SC, which increased FOMO, consecutively associated with a higher tendency for SNS addiction.

**Table 2:** Model 1: indirect, direct, and total effects.

Pathway	Estimate	SE	*Z*	*p*
Indirect 1: SWB → SC → SNS ADD	−0.02	0.01	−1.64	0.10
Indirect 2: SWB → FOMO→SNS ADD	−0.03	0.02	−2.29	0.02
Indirect 3: SWB → SC → FOMO→SNS ADD	−0.02	0.01	−2.30	0.02
Direct	−0.18	0.05	−3.59	0.00
Total	−0.26	0.05	−5.01	0.00

SC, social comparison; FOMO, fear of missing out; SNS ADD, SNS addiction.

H2 predicted that the mediating effect of SCA (Model 2) would be significant but not that of SCO (Model 3). As seen in Figures 3, 4, path A1 (Model 2: SWB × SCA; Model 3: SWB × SCO) was significantly negative for Model 2 (𝛽 = −0.21, *p* < 0.001) but not significant for Model 3 (𝛽 = −0.04, *p* = 0.513), showing that lower SWB was related to higher engagement in SCA but not SCO. Path A2 (Model 2: SCA × SNS addiction; Model 3: SCO × SNS addiction) was not significant for Model 2 (𝛽 = 0.09, *p* = 0.171) and Model 3 (𝛽 = 0.10, *p* = 0.077). Path D (Model 2: SCA × FOMO; Model 3: SCO × FOMO) was positive for Model 2 (𝛽 = 0.51, *p* < 0.001) and Model 3 (𝛽 = 0.45, *p* < 0.001), indicating that higher engagement in SCA, as well as in SCO was related to higher FOMO. Path B1 (SWB × FOMO) was not significant for Model 2 (𝛽 = −0.09, *p* = 0.063) but was negative for Model 3 (𝛽 = −0.18, *p* < 0.001), indicating that higher SWB was only associated with higher FOMO when SCO was present. The relationship between FOMO and SNS addiction (Path B2) was positive for Model 2 (𝛽 = 0.33, *p* < 0.001) and Model 3 (𝛽 = 0.32, *p* < 0.001), showing that higher FOMO was associated with higher levels of SNS addiction, regardless of SCA or SCO being present. The relationship between SWB and SNS addiction (Path C) was negative for Model 2 (𝛽 = −0.17, *p* < 0.001) and Model 3 (𝛽 = −0.19, *p* < 0.001), also indicating that regardless of the presence of SCA or SCO, lower SWB was associated with higher levels of SNS addiction.

As shown in Tables 3 and 4, the indirect effect 1 (Path A1 × Path A2) was not significant for Model 2 (𝛽 = −0.02, CI = [−0.05, 0.00], *p* = 0.203) and Model 3 (𝛽 = 0.00, CI = [−0.02, 0.00], *p* = 0.580). The indirect Effect 2 (Path B1 × Path B2) was not significant for Model 2 (𝛽 = −0.03, CI = [−0.05, 0.00], *p* = 0.085) but significant for Model 3 (𝛽 = −0.06, CI = [−0.08, −0.02], *p* = 0.003), indicating that lower SWB was associated with higher FOMO, which was consecutively associated with higher SNS addiction, but only if SCO was present. Confirming H2, the indirect Effect 3 (Path A1 × Path D × Path B2) was significant for Model 2 (𝛽 = −0.04, CI = [−0.05, −0.01], *p* = 0.004) but not for Model 3, (𝛽 = −0.01, CI = [−0.02, 0.01], *p* = 0.527), indicating that lower SWB was associated with higher SCA, which contributed to the increase of FOMO, consecutively associated with a higher tendency for SNS addiction.

**Table 3:** Model 2: indirect, direct, and total effects.

Pathway	Estimate	SE	*Z*	*p*
Indirect 1: SWB → SCA → SNS ADD	−0.02	0.01	−1.40	0.16
Indirect 2: SWB → FOMO→SNS ADD	−0.03	0.02	−1.78	0.08
Indirect 3: SWB → SCA → FOMO→SNS ADD	−0.04	0.01	−3.09	0.00
Direct	−0.17	0.05	−3.42	0.00
Total	−0.26	0.05	−5.00	0.00

SCA, social comparison of abilities; FOMO, fear of missing out; SNS ADD, SNS addiction.

**Table 4:** Model 3: Indirect, direct, and total effects.

Pathway	Estimate	SE	*Z*	*p*
Indirect 1: SWB→SCO→SNS ADD	0.00	0.01	−0.62	0.53
Indirect 2: SWB→FOMO→SNS ADD	−0.06	0.02	−3.19	0.00
Indirect 3: SWB→SCO→FOMO→SNS ADD	−0.01	0.01	−0.65	0.51
Direct	−0.19	0.05	−3.78	0.00
Total	−0.26	0.05	−5.00	0.00

SCO: social comparison of opinions; FOMO: fear of missing out; SNS ADD: SNS addiction.

## 4. General discussion

Overall, our results showed that the relationship between subjective well-being and SNS addiction was jointly mediated by social comparison and FOMO. The lower level of subjective well-being was associated with increased engagement in social comparison, which was associated with the increased level of FOMO, ultimately contributed to higher levels of SNS addiction.

In line with previous research (Burnell et al., 2019; Reer et al., 2019), the tendency to engage in social comparison was found to be closely associated with FOMO and mediated the relationship between subjective well-being and FOMO. Consequently, frequent upward social comparison might result in amplified FOMO as a feeling that others have more rewarding experiences than oneself. Our results also showed that FOMO fully mediated the relationship between social comparison and SNS addiction supporting the claim that one of the reasons for frequent social comparison resulting in SNS addiction might be *via* FOMO.

Most importantly, we tested the role of two different types of social comparison (ability and opinion) as a joint mediator with FOMO. Our results showed that when including the ability comparison as a social comparison variable, the joint mediating role of social comparison was the strongest (effect estimate = −0.04). In contrast, when including opinion comparison as a social comparison variable, the jointly mediating role of social comparison disappeared, confirming our hypotheses.

A possible explanation for the different roles played by ability and opinion comparisons might stem from distinct functions each type of comparison has in SNS contexts. First, as users mainly post visual contents, SNS might be a suitable platform for sharing pictures and videos directly relating to one’s skills, possessions, and physical traits, whereas one’s opinions or attitudes are rather indirectly portrayed in visual contents. Second, ability and opinion comparisons might guide SNS users to perceive the comparison target in a different way. When engaging in ability comparisons, the comparison target might be perceived as a competitor, but when engaging in opinion comparisons, the comparison target might be perceived as someone who shares the same or different beliefs (Park and Baek, 2018). When the comparison target is seen as an out-performing competitor, negative emotions such as the feeling of inferiority, envy, and the impression that others have better lives than oneself can be evoked. For the reason that such emotions serve as key elements of FOMO (Yin et al., 2021), future research should focus on whether those negative emotions stemmed from engaging in ability comparisons play a crucial role in explaining a potential vicious circle of subjective well-being, ability comparison, FOMO, and SNS addiction.

It is also important to note that the directionality of the relationship between subjective well-being and SNS addiction is not clear. Reer et al. (2019), for example, proposed a model with the same direction as the present study, while Burnell et al. (2019) proposed the opposite direction (SNS use leading to subjective well-being), and in both lines of research, the directionality was not clearly confirmed. Other researchers have argued that the relationship between subjective well-being and SNS use might be bidirectional (Wang et al., 2018). Indeed, people who engage in SNS activities in an effort to increase their life-satisfaction might contrarily experience decreased well-being due to the negative emotions evoked by making ability comparisons. In turn, people who experienced lower life-satisfaction might turn their attention to SNS platforms for a way to boost well-being. Due to the nature of the cross-sectional design, our data could not test this possibility but future research using a longitudinal design to verify a potential cyclic relationship is highly warranted.

Another limitation of the present study is that our sample was not a clinical population. Examining the joint mediating role of ability comparison and FOMO on the relation between subjective well-being and SNS addiction in those with a high SNS addiction tendency might offer a fuller picture of the behavioral mechanism. Comparing clinical and non-clinical samples might also verify whether distinct mechanisms relevant to social comparison constructs underlie in SNS behavior.

Despite the limitations, our study extends the theoretical framework of SNS behavior in relation to social comparison theory. Based on our findings, we argue that separate components of social comparison orientation differentially contribute to SNS addiction and therefore should be considered as distinct mechanisms explaining SNS addiction. A higher tendency for engaging in social comparison of ability, due to its self-evaluative nature, is more closely associated with experiencing negative consequences after engaging in upward comparisons in SNS platforms whereas a higher tendency for engaging in social comparison of opinion, due to its opinion validatory nature, is less likely to evoke negative emotions such as FOMO, envy, and depressive feelings. Likewise, future research is highly warranted to investigate more boundary conditions in regard to the directionality of social comparison (Rose et al., 2023), and social closeness of social comparison targets (Lup et al., 2015) to complement the existing theoretical framework of social comparison and SNS addiction.

Our findings also provide some implications on how to support people who suffer from excessive SNS use. First, users might be advised to actively avoid contents that might increase chances to engage in ability comparisons by regulating their SNS contents using filters and such, to minimize contents that show ability comparison materials (e.g., physical appearance, skills, performance) and maximize contents that focus more on sharing and comparing opinions and attitudes. Second, reassessing how the comparison target is viewed so not to perceive comparison targets as a competitor might help SNS users to avoid negative feelings (e.g., envy). When the comparison target is viewed as someone to learn from, social comparison might serve as an inspiration. Based on our findings, shifting one’s comparison process away from ability to opinion comparison might help reduce negative feelings and facilitate a controllable SNS use. Accordingly, focusing more on opinion comparison might also reduce the level of FOMO as negative emotions evoked by engaging in ability comparisons are closely linked to FOMO (Yin et al., 2021).

Our findings demonstrated the mediating roles of social comparison and FOMO in the relation between subjective well-being and SNS addiction while separately examining the role of ability and opinion social comparisons. The present research emphasizes the importance of dividing the social comparison components when investigating SNS behavior. Our data suggest that the tendency to engage in ability rather than opinion comparisons might play a bigger contributing role to SNS addiction and that FOMO in SNS contexts may be based more on the process of ability than opinion comparisons. As the distinctive roles of ability and opinion comparisons are often neglected by researchers (c.f., Kim et al., 2021), investigating further dissociable and overlapping roles of ability and opinion comparisons in the context of SNS behavior is highly recommended for future research.

## Data availability statement

The raw data supporting the conclusions of this article will be made available by the authors, without undue reservation.

## Ethics statement

The studies involving human participants were reviewed and approved by Departmental Review Board, Institute of Occupational, Economic, and Social Psychology, University of Vienna. The patients/participants provided their written informed consent to participate in this study.

## Author contributions

PS and HK designed the study, interpreted the results, and revised the manuscript together. PS collected and analyzed data and wrote the original manuscript. HK supervised data collection and analyses. All authors contributed to the article and approved the submitted version.

## Conflict of interest

The authors declare that the research was conducted in the absence of any commercial or financial relationships that could be construed as a potential conflict of interest.

## Publisher’s note

All claims expressed in this article are solely those of the authors and do not necessarily represent those of their affiliated organizations, or those of the publisher, the editors and the reviewers. Any product that may be evaluated in this article, or claim that may be made by its manufacturer, is not guaranteed or endorsed by the publisher.
